# Interdisciplinary Tasks in the Cyclotron Production of Radiometals for Medical Applications. The Case of ^47^Sc as Example

**DOI:** 10.3390/molecules24030444

**Published:** 2019-01-26

**Authors:** Alessandra Boschi, Petra Martini, Valentina Costa, Antonella Pagnoni, Licia Uccelli

**Affiliations:** 1 Department of Morphology, Surgery and Experimental Medicine, University of Ferrara, Via Luigi Borsari, 46-44121 Ferrara, Italy; petra.martini@unife.it (P.M.); licia.uccelli@unife.it (L.U.); 2 Legnaro National Laboratories, Italian National Institute for Nuclear Physics (LNL-INFN), Viale dell’ Università, 2, 35020 Legnaro (PD), Italy; 3 Department of Chemical and Pharmaceutical Sciences, University of Ferrara, Via Luigi Borsari, 46-44121 Ferrara, Italy; valentina.costa@unife.it (V.C.); antonella.pagnoni@unife.it (A.P.)

**Keywords:** radiometals, cyclotron, target, automation, radiochemical separation

## Abstract

The growing number of cyclotrons of different sizes installed in the territory has given a strong impulse to the production of conventional and emerging radionuclides for medical applications. In particular, the great advantage of using medical cyclotrons is the possibility to produce on-site, when needed (on-demand), with medical radionuclides of interest encouraging the personalized medicine approach. Radiometals satisfy the ideal characteristics that radionuclides should have for routine employment in nuclear medicine, especially since they have a robust chemistry suitable to synthetize stable in vivo radiopharmaceuticals with high radiochemical yields. In this letter several interdisciplinary aspects involved in the radiometals cyclotron production cycle are summarized focusing the attention on cyclotron production facilities, target material, and chemical processing available for medical applications. As an example, the current status and recent development in the production of the theranostic radionuclide scandium-47 have been reported.

## 1. Introduction

Nuclear medicine (NM) is based on the use of radiopharmaceuticals, pharmaceutical drugs containing one or more radioactive nuclides, to investigate different kind of diseases such as heart disease, neurological, endocrine, gastrointestinal disorders and other anomalies within the body, metabolic processes, or to treat tumors. Moreover, NM is considered as a part of molecular imaging since, by using a wide range of different emitter radionuclides and radiopharmaceuticals, it provides pictures of what is happening inside the body at the molecular and cellular level with the aim to study specific biological process including pathophysiology of certain diseases [1,2,3,4]. There are two kinds of radionuclides involved in diagnostic procedures: those that directly decay by emitting γ rays, suitable for conventional NM scans, such as single-photon emission computed tomography (SPECT), and those that decays by β^+^ emission which subsequently undergoes annihilation with an electron emitting two gamma rays at 511 keV each, suitable for positron emission tomography (PET). In nuclear medicine, it is also possible to perform therapeutic treatments of tumors employing nuclides having high energy β^−^ or α emission able to destroy cells responsible for pathologies thanks to the biological effects of the emitted radiation [1,5,6]. A new frontier of nuclear medicine is in theranostics, a medical approach introduced by Herzog et al. [7,8] in 1993, combining therapeutic and diagnostic effects by using radionuclides with proper decay radiations (single-element theranostics radionuclides e.g., ^47^Sc: T_1/2_ = 3.3492 days, E_γ_ = 159.381 keV and E_β-mean_ = 162.0 keV; multiple-element theranostics radiopharmaceuticals e.g. ^188^Re: T_1/2_ = 17.004 h, E_β-mean_ = 763 keV with ^99m^Tc: T_1/2_ = 6.0072 h, E_γ_ = 140.511 keV [9]). Theranostics provides a personalized medicine approach allowing the selection of patients that will benefit from the particular treatment, also avoiding unnecessary and expensive therapies [8,10,11,12]. 

The choice of the appropriate radionuclide is based not only on its nuclear and chemical properties but also on production easiness, costs, and prompt availability. In general, nuclides employed for diagnostic procedures should: (1) have a half-life long enough to allow the radiopharmaceutical preparation but short enough to minimize the adsorbed patient dose (in general less than 24 h); (2) emit only low energy gamma radiations or β^+^ particles, suitable for SPECT or PET imaging respectively; (3) have a robust chemistry and chemical properties suitable to synthetize radiopharmaceuticals with high radiochemical yields and particularly stable in vivo. Unfortunately, there are no nuclides able to completely satisfy all those characteristics and, therefore, only few radionuclides are routinely employed in NM. Those that mostly show similar characteristics to those indicated above are metals. Thanks to their rich coordination chemistry, transition metals offer a wide range of opportunities to link with different ligands in radiopharmaceutical preparations.

Radiometals can be produced by different methods: nuclear reactors, generators, and cyclotrons, each of them having advantages and disadvantages. Nuclear reactor is a centralized production method characterized by high production efficiency, which guarantee the supply to a large territory, but presents as main disadvantages the high investment and operational costs and the production of large amount of long-lived radioactive waste, in addition to public safety issues. 

The production of radionuclides by cyclotron offers several advantages, including high specific activity of the produced radionuclide, decentralized production easily programmable, smaller production of long-lived radioactive waste than in nuclear reactors, and smaller investment. In particular, the technological advancement in the cyclotron-based radionuclides production sector has given a strong impetus to the use of radiometals in medical applications [13,14,15].

The radionuclide generator systems intrinsically need an appropriate long-lived parent radionuclide, on which the short-lived daughter radionuclide production is based, and its in-house use depends on the timed elution cycles [14]. 

The scope of this letter is to provide a short overview on the different interdisciplinary and tightly connected aspects involved in the cyclotron production of radiometals for nuclear medicine. A particular section will be dedicated to the current status and recent development in the production of the theranostic radionuclide ^47^Sc.

## 2. Cyclotron Production Facilities

Among particle accelerators, cyclotron is the most frequent choice for radionuclides production [15]. Cyclotrons can accelerate different particles such as protons, deuterons, and alphas at variable energies originating nuclear reactions making possible the production of a wide variety of medical isotopes. Main advantages of using accelerators for the production of medical radionuclides lies in the high specific activity (SA) achievable, the smaller amount of radioactive waste generated compared to nuclear reactors, the cost-effectiveness and scalable on-demand daily availability of the radionuclides.

The growing number of cyclotrons of different sizes [16] installed on the territory is in line with the constant expansion of radionuclides needs for Nuclear Medicine applications. Small cyclotrons (proton energy, Ep < 20 MeV), whose number around the world in the last ten years has practically doubled, are the tool of choice for the in-hospital production of most PET isotopes routinely involved in diagnostic investigations [17]. They are located in regional centers for the production of short half-life PET radionuclides in sufficient amount to cover the hospital needs and many of them have a multiple targets capability on two or more extracted beamlines [18]. Medium cyclotrons, with energies between 20 to 35 MeV, are usually installed in academic research institutes or commercial facilities specialized in production of intermediate half-life SPECT and PET radionuclides. Large multi-purpose research cyclotrons, with energies greater than 35 MeV and up to 500 MeV, are commonly located in academies and governments research institutes and are mostly used for the production of therapeutic radionuclides, parent nuclides to be loaded onto generator systems and for research [18]. 

A wide selection of radiometals can be produced by cyclotron. In particular, small medical cyclotrons, are currently producing, routinely or on research scale, ^99m^Tc, ^124^I, ^89^Zr, ^64^Cu, ^67^Ga, ^68^Ga, ^86^Y, ^44^Sc, and ^111^In [19]. Increasing the beam energy to 30-35 MeV nuclear reactions gives access to a large number of isotopes such as ^201^Tl, ^67^Ga, ^111^In and ^64^Cu; while cyclotron that accelerate protons up to around 70 MeV can also produce ^82^Sr, ^68^Ge, ^67^Cu, and ^47^Sc [20]. In Table 1 a list of “cyclotron-produced” radiometals and some of possible nuclear reactions are reported.

## 3. Radiometals Cyclotron-Production Process 

**Figure 1:**
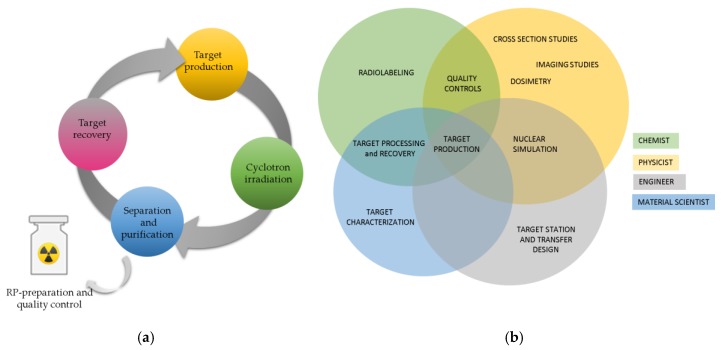
(**a**) Radiometal cyclotron production cycle; (**b**) Involved competence and interrelated tasks and contributions.

Several interdisciplinary tasks and expertise are necessary to accomplish the production route of radiometals by means of cyclotrons. The theoretical knowledge of the nuclear reaction cross-sections involved in the production, for example, is required in the choice of the optimal irradiation parameters, that usually lies in a compromise between production yield and quality parameters such as isotopic purity (IP) and radionuclidic purity (RNP). Competence in engineering, chemistry, and material sciences are fundamental in the choice of a dedicated target (either solid, liquid, or gas target), in the development of a highly efficient separation/purification procedure in order to achieve high radiochemical purity (RCP) and RNP products, and in the development of a target recovery process to minimize production costs. In Figure 1a a schematic representation of a radiometal cyclotron production cycle for medical application is reported. Moreover, the radiometal cyclotron production must be supported by quality controls to evaluate the injectability and quality of produced metal used then for the preparation of final radiopharmaceutical product. Figure 1b pointed the diverse nature of involved competence and interrelated tasks and contributions.

In the following section the principal technological aspects of the radiometal cyclotron production cycle will be discussed.

### 3.1. Solid and Liquid Targets 

**Figure 2:**
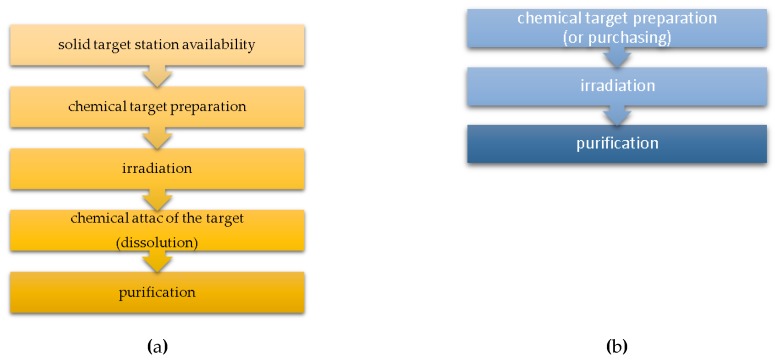
Radiometal cyclotron production approach through solid target (**a**) and liquid target (**b**).

Mainly two different kind of targets can be used for radiometals cyclotron production: liquid and solid targets. There is no example of a gas target used for radiometals cyclotron production reported in the literature presently. Liquid targets have the advantage to be simple to handle and to produce, to allow a faster post-irradiation process, avoiding long dissolution procedure, and to be less expensive than solid configuration, since less material is involved. Contrariwise, solid targets, despite more expensive due to the high amount of enriched target material involved, allow higher production yields but requires more post-irradiation chemical separation steps (Figure 2). Moreover, hospital-based cyclotron facilities, solid targets require technological and structural investment for specific target station and more complex automated delivery systems. The cyclotron production of various positron-emitting radiometals, such as ^86^Y, ^94m^Tc, ^89^Zr, ^61^Cu, ^68^Ga, and ^44g^Sc, using a liquid target has been intensively investigated in the past ten years [21,22,23,24,25,26,27,28,29,30]. Several problems correlated with the use of liquid targets were reported. In particular, the corrosiveness of the metal acidic salt solutions, degrading the internal surface of target body and the HAVAR® foil; the development of gas during the irradiation, determining an overpressure in the target; and salt precipitation [21,22,30]. To overcome these problems, the following solutions have been proposed:

→ substitution of the aluminum target body with one made of niobium, more resistant to corrosion;
→ usage of nitrate rather than chloride solution thus minimizing gas formation and avoiding HAVAR^®^ corrosion;
→ addition of nitric acid to the target solution thus preventing the formation of solid precipitate and minimizing gas evolution;
→ substitution of the HAVAR® foil with a niobium foil improving the resistance of the foil to the acidity of the target solution.

In the case of scandium-44, the use of liquid targets improved its availability for tracer development. This radiometal can be produced via ^44^Ca(p,n)^44g^Sc reaction by irradiating, with a 13 MeV proton-beam cyclotron on a standard liquid target station, a liquid target solution containing a high concentration of natural-abundance calcium nitrate tetrahydrate (Ca(NO_3_)_2_ · 4 H_2_O) [24]. Alves et al. [28,29] have recently demonstrated that the production of ^68^Ga, ^64^Cu, and ^61^Cu through irradiation of liquid target allows to produce high specific activity of these radionuclides, reducing cost and processing time in comparison with cyclotron production through the solid target approach. The cyclotron production of ^68^Ga is particularly interesting as it could economically strongly compete with the generator supply chain: <25.000 €/year including the ^68^Zn target recovery, a quarter of the generator-production costs [30]. Recently, Alves at al. [28] produced, with the liquid target technology, 6 GBq of ^68^Ga with a IBA 18/9 Cyclone cyclotron. Moreover, Riga et al. (2018) have also demonstrated to be able to produce directly in hospital, 4 GBq (at EOB) of ^68^Ga with a GE PETtrace cyclotron [30].

Solid targets, in the form of metallic foil/coin, metallic powder or in oxide form, are mainly used for the cyclotron production of ^99m^Tc, ^67^Ga, ^111^In, ^64^Cu, ^61^Cu, ^68^Ge, ^103^Pd, and ^89^Zr. For example, highly enriched ^100^Mo targets are used for the direct cyclotron ^99m^Tc production, via the ^100^Mo(p,2p)^99m^Tc nuclear reaction while cadmium solid targets, highly enriched in the isotope 112, are commonly used for the cyclotron production of indium-111 via ^112^Cd(p,2n)^111^In nuclear reaction [31,32]. 

In Table 2 different type of target used for the cyclotron production of the selected radiometals are reported.

### 3.2. The role of Radiochemical Processing in the Radiometal Cyclotron-Production

With the aim to obtain an injectable radiopharmaceuticals labelled with radiometals, the radiochemical processing of an irradiated target is a fundamental step in the cyclotron production. The process should also aim at recovering the enriched target material, that in general is very expensive. A radiochemical procedure should be fast, allow high recovery yield, high chemical, radiochemical and radionuclidic purity, high SA and sterility. To minimize human errors, ensure high process reproducibility and operators radiation protection the use of remotely controlled automatic systems is recommended [13,29,46,48,49].

The choice of a radiochemical processing method, that can be applied for the extraction/separation and purification of a medical radiometal should take into account: (1) physical and chemical properties of the target material (e.g. liquid or solid); (2) physical and chemical properties of the desired radiometal produced in the target during the irradiation; (3) quality requirements of the final product for the human use (ruled by the Pharmacopoeia by means of specific monographs); and (4) quality requirements of the final product for specific radiolabeling process (ruled by pharmaceutical manufacturer in case of kit labeling procedure) [50].

In the case of solid targets, the radiochemical processing requires first a dissolution step of the target and, if necessary, further chemical treatments to convert the dissolved target into proper chemical species to be efficiently involved in the separation procedure [51]. In the case of liquid targets, the dissolution step is not necessary and only the chemical conversion to proper species may be requested. Further purification steps could be necessary using liquid targets since the dissolution of some materials coming from either the vacuum isolation foil or from the target body, resulting in the contamination with radioactive or stable metals salt, ions, etc., of the target solution may occur during the irradiation [51].

One or a combination of conventional separation methods such as chromatography, solvent extraction (SE), precipitation, distillation, etc., can be selected to isolate the desired radionuclides and eliminate radioactive contaminants. 

**Figure 3:**
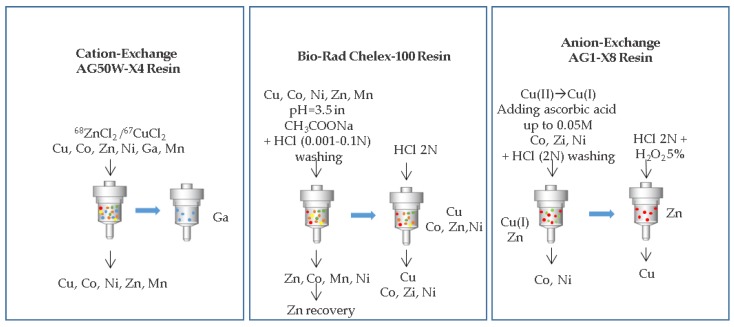
Schematic representation of the separation of copper isotopes from zinc and gallium contaminants reported by Medvedev et al. [53].

Ion exchange chromatography is one of the most commonly used methods for radiometals separation in nuclear medicine radiopharmacies since it is readily adaptable to automation [15,49]. A pratical example is use for the purification of generator eluted gallium-68 for the preparation of ^68^Ga-DOTA-TOC [49,52]. Ion exchange chromatography is based on the distribution of an element between the mobile and solid phase depending on the ionic form, the solute concentration and the functional group on the resin. Anions are involved in the exchange when the functional groups of the solid phase are positively charged and, conversely, cations are involved when they are negatively charged. Effectiveness of the process can be modulated by variables such as ionic concentration, column volume and diameter, flow rate and eluents. This technique is very useful for separating radionuclides from the bulk target and is for example applied for the separation of copper isotopes from zinc and gallium contaminants (Figure 3).

A particular type of chromatography, aqueous biphasic extraction chromatography (ABEC), was chosen for the separation of cyclotron produced ^99m^Tc as described by Schaffer et al. [43]. This method is based on the use of a hydrophobic polyethylene glycol (PEG)-based column resins, that immobilizes the ^99m^Tc-pertechnetate leaving the molybdate flowing through. 

The solvent extraction (SE) is a different separation method that can be efficiently applied for the separation of radiometals. For example SE allows the efficient separation of cyclotron produced ^99m^Tc after irradiation of metallic molybdenum metal target enriched in the isotope molybdenum-100 [54]. This method is based on the selective extraction by methyl ethyl ketone of ^99m^Tc, in the chemical form of [^99m^Tc]TcO_4_^-^, from an alkaline solution containing pertechnetate, molybdate, and by products, in high yield and high quality. The SE of metals by an organic solvent, such as in the case of ^99m^Tc, is often followed by further purification step of the product from the solvent, by means of column chromatography [55,56,57,58]. This method allows to obtain [^99m^Tc]TcO_4_^-^ with high chemical and radiochemical purity suitable for the following radiolabeling procedure, particularly sensitive to the quality of ^99m^Tc-pertechnetate solution [13,59,60]. 

#### Automation

The entire automation of a radiochemical separation or a radiopharmaceutical synthesis is required for clinical application where the amount of radioactivity is high [61]. Commercial or custom-made modules are routinely involved in radiopharmacy practice for the preparation of small-scale radiopharmaceuticals (synthesis, dilution, purification, filtration, dispensing, etc.) [62]. Sector companies provide different customizable modules design suitable for the processing of radioactive materials together with a wide selection of consumables to personalize your own automatic procedure [52,55,62].

The module assembly consists in a mechanical and a chemistry part [62]. The building block of the module and other physical devices provided by the manufacturer, representing the mechanical part, usually are: (1) valves block: valves can be of different type (solenoid, pinch, pneumatic, rotary valves) and they can be two or three way valves made of different materials; (2) pumps: syringe, peristaltic and vacuum pump are the most used in this field; (3) gas flow controller or/and pressure regulator: as alternative way to perform liquid transfer; (4) reactor heater and fast cooling feature; and (5) detection instrumentation: pressure, UV, radiation detectors. The chemistry part, instead, is an assembly of vials, tubing, fittings, etc., in which reagent motion allows for a sequence of chemical operations and reactions to obtain the desired final product. This part can be pre-assembled by the manufacturer and provided as disposable device, called a cassette, or be non-disposable in permanent contact with the mechanical part. Mechanical and chemistry parts are connected and commanded by PLC (programmable logic controller), PC and graphical editing software for programming the sequence of the procedure together with a scheme of the fluid path as user interface. 

The automation process of a separation and purification procedure is the final step of the process optimization. 

Analytical characterization techniques, such as ICP-MS, ICP-OES, MP-AES, gas chromatography, etc., allow for the identification of the proper procedure, usually a combination of separation techniques aiming at separating and purifying the desired radionuclide from the target, other contaminants and solvents. Subsequently, a semi-automatic module can be designed as intermediate step useful when performing low activity experiments to optimize and define a continuity of the operations. Finally, the complete automation allows for: the reproducibility of the results, the minimization of activity losses and operation time, the maximization of the final recovery yield, the production under GMP rules. 

### 3.3. The Target Recovery

The target recovery and its recycling is a very important step to complete the radiometal production cycle and make it economically sustainable. In most cases the production of radiometals by cyclotron require the use of highly enriched target material to achieve high radionuclidic and isotopic purity of the product. This is the case of ^64^Ni, used for the production of ^64^Cu, or ^100^Mo used for the ^99m^Tc production by cyclotron, where the recovery and recycling of the target is very important to minimize the production costs. On the contrary there are cases for which recovery is not necessary, as for ^nat^Zn target that is quite inexpensive [29].

A deep evaluation of the metal target recovery strategy needs to be done in order to keep the radiometal production costs within affordable limits and the quality parameters of the final product, from recycled target, within the pharmacopoeia limits. The chemical strategy to choose for the recovery is strictly dependent on the chemical form of the target material and the method used for the target production. 

The most complex case in literature is represented by the metallic molybdenum-100 recovery after separation and purification of the direct cyclotron produced ^99m^Tc. The molybdenum recovery in the metallic form needs several steps to first decompose the molybdate anions into trioxide MoO_3_ form, then to reduce the oxide in the metallic form by a multi-step hydrogen reduction. Gagnon, et al. [63] used a two-step reduction procedure: an exothermic conversion of MoO_3_ to MoO_2_ with a ramp rate between 500 and 750 °C in low concentration H_2_ gas atmosphere as a first step, while the second step consists in the reduction of MoO_2_ to Mo metal up to 1100 °C in H_2_ 100% atmosphere. To evaluate the reduction efficiency and the isotopic composition, XRD (X-ray diffraction) and inductively coupled plasma mass spectrometry (ICP-MS) characterization techniques need to be applied to the recycled sample. Gagnon et al. [63] reported a complete recovery cycle achieving an overall metal to metal yield of 87%.

On the contrary, the recovery of metals that can be conveniently treated for electroplating results more simple to perform. Electrolytic cells can be integrated in the automated unit used for the delivery/recovery of the metal by pneumatic systems. Matarrese et al. [64] reported a procedure for dynamic Ni electroplating with recirculation of the electrolytic solution in a well-defined volume of a cylindrical chamber, integrated in an automated module for Cu radioisotope production.

## 4. The case of Cyclotron Produced ^47^Sc: an Example

Scandium-47 (T_1/2_ = 3.35 d) is particularly interesting for radio immunotherapy of small tumors and cancer metastases due to its suitable *β*^−^ emission (E_βav_ = 162.0 keV). Together with ^44^Sc (or ^43^Sc, it represents the ideal matched pair for theranostic applications [65]. Moreover, by exploiting its γ-emission at 159 keV (68.3%), this radionuclide can be used as single theranostic radionuclide performing SPECT imaging studies. From the chemical point of view Sc(III) presents similar coordination chemistry to Lu(III) and Y(III), allowing the use of ligands already developed for two well-established radionuclides, ^177^Lu (T_1/2_ = 6.647d, E_βav_ = 134.2 keV) and ^90^Y (T_1/2_ = 64.0 h, E_βav_ = 933.6 keV) [9,66,67].

The critical issue in the use of ^47^Sc is the lack of availability in sufficient amount and at reasonable cost. Different routes have been explored in the past for the ^47^Sc production (Table 3). These are mainly based on the use of nuclear reactor and neutrons irradiation (*E*_n_ > 1 MeV) of ^47^TiO_2_ target via the ^47^Ti(n,p)^47^Sc reaction, or thermal neutron irradiation of target enriched in the isotope ^46^Ca via the ^46^Ca(n,γ)^47^Ca (T_1/2_ = 4.5 d, β^−^) →^47^Sc nuclear reaction [66]. This latter method presents the advantage of using a ^47^Ca/^47^Sc generator, but the disadvantage of the highly cost ^46^Ca target material. Alternative production ways are based on proton or gamma irradiation of ^nat^Ti or ^48^Ti in electron linear accelerator with a lower efficiency in comparison with the nuclear reactor production. A new production way based on cyclotron has been recently proposed. Minegishi et al. [67] reported the cyclotron production of ^47^Sc based on the alfa-irradiation of enriched ^44^Ca target. Sadly, the low cross-section of the ^46^Ca(α,p)^47^Sc reaction does not allow to produce high amount of the desired radiometal necessary to perform clinical applications.

Khandaker et al. [68], reported a dataset for the ^nat^Ti(p,x) ^43,44m,44g,46,47,48^Sc production leading to various practical applications. In this study they demonstrated that optimum production, 12 MBq/mA h, of ^47^Sc can be achieved by irradiating natural titanium target over the energy range 33-22 MeV with only ^46,48^Sc impurity of about 3%. Moreover, using lower energy (<33 MeV) cyclotron and enriched ^50^Ti target, the ^47^Sc production in large amount minimizing the co-production of ^46,48^Sc radionuclides could be possible.

From these considerations it is obvious that, the work of the physicist dedicated to study the most promising nuclear reactions, by measuring unknown cross sections, optimizing the irradiation parameters considering the co-production of contaminant radionuclides, became very helpful to respond to the increasing request of ^47^Sc production for clinical applications. In fact, while non-isotopic impurities in irradiated target can be removed by optimized chemical separation processes, isotopic impurities (e.g., ^xz^Sc) can be minimized only using enriched isotopes as target materials and/or by carefully selecting the effective particle energy range in the target. 

After the ^47^Sc cyclotron production, the desired product has to be separated and purified from the target. In this field, skills in radiochemistry and analytical process are needed to design and optimize a radiochemical method to isolate ^47^Sc in a chemical form suitable for radiolabeling. Variety of ^47^Sc separation methods from metallic Ti, TiO_2,_ and CaCO_3_ targets based on solvent extraction by tri-n-butyl phosphate (TBP), extraction chromatography or ion exchange processes has been reported [66,68,69,70].

In the case of calcium targets, all proposed method are simple, fast and allow to recover high percentage of the desired radionuclide and to apply simple chemical procedure for recovery [20]. In the case of titanium targets, the proposed procedure are longer and sometimes requiring time consuming dissolution and evaporation steps because of the greatest difficult in dissolving the titanium in particular in the TiO_2_ form. Moreover, in the greatest part of the proposed procedures, the use of fluoridric acid is required and accordingly the employment of proper and resistant material to this acid is necessary [71].

Recently some procedures have been developed to dissolve titanium in metallic form using hot HCl solution (8 M), avoiding the use of fluoridric acid, and separate ^43^Sc from ^46^Ti using a DGA extraction chromatographic resin [70]. This resin selectively retains Sc, leaving Ti to flow through the resin, in a 4M HCl solution. Pourmant et al. [72] also reported the strong Sc(III) retention on DGA resin and the negligible Ti(III) retention at HCl molarities below 6 M. After elution with 4.0 mL of HCl 0.1 M scandium is concentrated in a smaller volume by the use of a second column containing SCX cation exchange resin and eluted with 4.8 M NaCl/0.13 M HCl as eluent.

**Figure 4:**
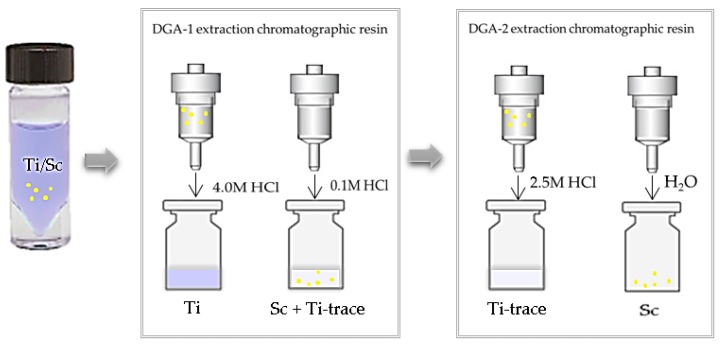
Schematic representation of the separation of scandium is from titanium using DGA-extraction chromatographic resin.

Our group [73] also recently investigated a similar procedure (to be published) that allow to obtain scandium in a water solution using two DGA resins (Figure 4). Shortly, we dissolved 10 mg of metallic ^nat^Ti in 3 mL of boiling 4.0 M HCl and to the resulting solution 2 µL (~2 µg) of a standard solution of scandium in HNO_3_ have been added. The solution was loaded onto a 1 mL column cartridge, containing ~87 mg DGA-N extraction chromatographic resin (normal, particle size 50-100µm, TrisKem International, Bruz, France). After resin washing with 5 mL of 4.0 M HCl to ensure complete removal of Ti(III), Sc(III) was eluted in 4 mL of 0.1 M HCl. The eluted scandium solution was rinsed with 1.5 mL of concentrate HCl to get a HCl solution at molarity around 2.5–3. The solution was loaded onto a second 1 mL column cartridge, containing ~30 mg DGA-N and after washing with 5.0 mL of 2.5 M HCl, Sc(III) was eluted with 0.7 mL of water. The use of the second resin allows to maximize the scandium/titanium separation yield and concentrate the final product in a solution compatible with radiolabeling procedures. All eluted fractions were analyzed by ICP-OES and no trace (Ti detection limit 0.2 ppm) of titanium in the final scandium solution (recovery yield > 95%) was found. This separation and purification procedure can be easily automated aiming to maximize the scandium isolation and recovery yield. 

With the aim to reduce the production costs, due particularly to the elevated prizes of the highly enriched material used for the target, the recovery of calcium and titanium is also an important part in the ^47^Sc cyclotron production process. Titanium recycling is generally performed at basic pH (~8), that can be reached with ammonia solution, following the precipitation of TiO_2_ [67], while the recovery of calcium target can be performed though the precipitation of Ca-oxalate at pH of 4.5–5 with subsequent conversion to carbonate by slowly heating up to 500 °C [74].

## 5. Conclusions

An overview on the different tasks involved in the radiometals production by cyclotron has been reported. It is clear form the above discussion is clear that different disciplines, knowledge, and competences are necessary: nuclear physics, that with nuclear reaction excitation function studies give a crucial input to choose an optimum target irradiation conditions in order to maximize desired isotope production and minimize unwanted by-products; mechanical engineering and material sciences are needed to address issues of target heating, induced mechanical stress, and material compatibility of targetry; radiochemists are involved in particular in the targeting post-processing to separate and purify the desired final cyclotron-produced radionuclide from the bulk target and impurities. All are also involved in the recovery of the target material, fundamental point to make the economically sustainable production.

With the aim to reduce the production costs and to encourage the research on the investigation of different radiometals radiopharmaceuticals, some studies are actually ongoing on the development of production strategies based on the use of liquid target as for ^86^Y, ^89^Zr, ^61^Cu, ^68^Ga, and ^44^Sc. This strategy avoids the use of a specific target station for solid targets and the dissolution of irradiated material, reducing the time of post-processing procedures. Unfortunately, this approach can not be used for the main part of radiometals and needs more studies dedicated to increase the production yields. At the same time, high-performance solid target design optimization, aiming at maximizing the heat dissipation efficiency, is the main goal of most research groups in this field.

## Figures and Tables

**Table 1:** List of cyclotron-produced radiometals and some of possible nuclear reactions [9].

radionuclide	Life-time	Nuclear Reaction	Application
^61^Cu	3.33 h	^64^Zn(p,α)	PET
^64^Cu	12.7 h	^64^Ni(p,n)	PET
^67^Cu	61.9 h	^68^Zn(p,2p)	Therapy/SPECT
^67^Ga	78.3 h	^68^Zn(p,2p)	SPECT
^68^Ga	68 min	^69^Ga(p,2n)^68^Ge → ^68^Ga	PET
^82m^Rb	5 min	^85^Rb(p,4n) ^82^Sr → ^82m^Rb	PET
^44^Sc	3.97 h	^44^Ca(p,n)	PET
^47^Sc	79.2 h	^47^Ti(n,p) ^48^Ca(p,2n)	Therapy/SPECT
^99m^Tc	6h	^100^Mo(p,2n)	SPECT
^86^Y	14.7	^86^Sr(p,n)	PET
^103^Pd	17.5 d	^103^Rh(p,n)	Therapy
^111^In	67.2 h	^112^Cd(p,2n)	SPECT
^186^Re	90.6 h	^186^W(p,n)	Therapy/SPECT
^201^Tl	73.5 h	^203^Tl(p,3n)^201^Pb → ^201^Tl	SPECT
^89^Zr	78.4 h	^89^Y(p,n)^89^Zr	PET

**Table 2:** Type of target used for the cyclotron production of the selected radiometals.

Radionuclide	Target Type	Chemical form of the Target	References
^44^Sc	solid	Metallic calcium pellets, ^44^CaCO_3_ powder	[33,34]
	liquid	^44^Ca(NO_3_)_2_x·4H_2_O solution	[24]
^64^Cu	solid	^64^Ni(95% enrich.)	[35]
	liquid	^64^Ni(NO_3_)_2_x·6H_2_O solution	[29]
^67^Cu	solid	^s68^Zn, ^nat^Zn, ^70^Zn metal; ZnO	[29,36]
^67^Ga	solid	^68^Zn, ^nat^Zn, ^67^Zn metal	[37]
^68^Ga	solid	^68^Zn metallic	[19,38]
	liquid	^68^Zn(NO_3_)_2_x·6H_2_O solution	[21,28,30]
^82^Sr	solid	^nat^RbCl or ^nat^Rb metal	[39]
^86^Y	solid	^nat^SrCO_3_	[40]
	liquid	^nat^Sr(NO_3_)_2_ solution	[21]
^89^Zr	solid	^89^Y foil, pellets, Y_2_O_3_	[41]
	liquid	^nat^Y(NO_3_)_3_ · 6H_2_O solution	[21]
^99m^Tc	solid	^100^Mo metal	[13,42,43]
^103^Pd	solid	^103^Rh metal foil	[44,45]
^111^In	solid	^nat^Cd, enriched ^112^Cd or ^nat^Ag	[15]
^186^Re	solid	^186^WO_3_	[46,47]
^201^Tl	solid	^203^Tl metal	[15]
^203^Pb	solid	^nat^Tl, ^205^Tl metal	[15]

**Table 3:** Direct and indirect ^47^Sc production routes.

Direct Production	Indirect Production
^48^Ca(p,2n)^47^Sc	^48^Ca(p,x)^47^Ca → ^47^Sc
^46^Ca(α,p)^47^Sc	^46^Ca(n,γ)^47^Ca → ^47^Sc
^47^Ti(n,p)^47^Sc	
^48^Ti(p,2p)^47^Sc	
^49^Ti(p,x)^47^Sc	^49^Ti(p,3p)^47^Ca → ^47^Sc
^50^Ti(p,x)^47^Sc	^50^Ti(p,x)^47^Ca → ^47^Sc
^nat^V(p,x)^47^Sc	
